# [Retracted] Fangchinoline suppresses growth and metastasis of melanoma cells by inhibiting the phosphorylation of FAK

**DOI:** 10.3892/or.2024.8740

**Published:** 2024-04-22

**Authors:** Jie Shi, Bingyu Guo, Qiang Hui, Peng Chang, Kai Tao

Oncol Rep 38: 63–70, 2017; DOI: 10.3892/or.2017.5678

Following the publication of this paper, it was drawn to the Editor's attention by a concerned reader that certain of the Transwell migration and invasion assay data shown in Fig. 3C and D on p. 67 were strikingly similar to data appearing in different form in another pair of articles written by different authors at different research institutes, one of which (subsequently retracted) had already been published elsewhere prior to the submission of this paper to *Oncology Reports*, with the other having been submitted for publication at around the same time. In addition, duplications of data were identified within Fig. 3C and D, such that data which had been used to represent the results from differently performed experiments had apparently been derived from the same original source. Given that the abovementioned data had already apparently been published previously, the Editor of *Oncology Reports* has decided that this paper should be retracted from the Journal. After having been in contact with the authors, they accepted the decision to retract the paper. The Editor apologizes to the readership for any inconvenience caused.

